# Fab’ Fragment‐Immobilized Gold Surface for Capturing EpCAM‐Positive Breast Cancer Cells

**DOI:** 10.1002/elsc.70043

**Published:** 2025-09-12

**Authors:** Elif Kaga, Sadik Kaga, Ozlem Yalcin, Gizem Fatma Erguner, Nurullah Okumus

**Affiliations:** ^1^ Department of Medical Services and Techniques Afyonkarahisar Health Sciences University Afyonkarahisar Türkiye; ^2^ Department of Biomedical Engineering Afyon Kocatepe University Afyonkarahisar Türkiye; ^3^ Research Center for Translational Medicine (KUTTAM) Koc University Istanbul Türkiye; ^4^ Department of Pediatrics Afyonkarahisar Health Sciences University Afyonkarahisar Türkiye

**Keywords:** APTES, EpCAM, Fab’ fragment, thiol‐gold bond

## Abstract

**ABSTRACT:**

Circulating tumor cells (CTCs) are cancer cells present in the bloodstream that originate from primary or metastatic sites. Sensitive and selective capture of these rare cells is essential for early diagnosis, metastasis prevention, and prognosis prediction. In this study, we demonstrated the effectiveness of a surface functionalized with epithelial cell adhesion molecule (EpCAM) Fab’ (fragment‐antigen‐binding) fragments for the specific capture of EpCAM‐positive human breast cancer cells. EpCAM antibody Fab’ fragments were produced through pepsin digestion and characterized by SDS‐PAGE analysis. Glass surfaces were silanized before being coated with a thin layer of gold via sputtering to ensure stability. The Fab’ fragments were immobilized on the gold‐coated glass surfaces through strong gold‐thiol bonds. The modified surfaces were then characterized using Fourier‐transform infrared spectroscopy (FTIR), scanning electron microscopy (SEM), and atomic force microscopy (AFM) analyses. Cell capture performance was assessed using fluorescence microscopy with both EpCAM‐positive and EpCAM‐negative cell lines. The results show that the Fab’‐modified surface offers a promising platform for the selective immunocapture of EpCAM‐positive cells.

*Practical application:* This study presents a preliminary design of a Fab’ fragment‐immobilized surface for the selective capture of EpCAM‐positive breast cancer cells. The surface modification relies on spontaneous Au‐S bonding, offering a simple and effective chemical method. The modified surface demonstrates strong potential for integration into future biosensor platforms for detecting circulating tumor cells. Such a system is promising for advanced diagnostics, monitoring, disease progression, and personalized treatment uses.

## Graphical abstract

APTES modified glass surfaces enable stable gold coating. Anti‐EpCAM Fab’ fragments can be obtained from anti‐EpCAM antibody by consecutive pepsin digestion and TCEP reduction. The free ‐SH groups on anti‐EpCAM Fab’ fragments give strong bounds with Au elements on the gold surface. Obtained anti‐EpCAM Fab’ immobilized surface is capable of sensing and capturing EpCAM positive cells with high precision.

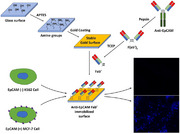

**Abbreviations**
AFM: atomic force microscopy
APTES: (3‐aminopropyl)triethoxysilane
CTC: circulating tumor cell
EpCAM: epithelial cell adhesion molecule
Fab’: fragment antigen‐binding
PBS: phosphate‐buffered saline
SDS‐PAGE: sodium dodecyl sulfate–polyacrylamide gel electrophoresis
SEM: scanning electron microscopy

## 1 Introduction

The main cause of breast cancer‐related deaths is complications that occur after metastasis in other tissues and organs [1]. Metastasis starts with the spread of cells originating from the primary tumor into the host tissue and continues with the spread of these cells to other tissues as a result of intravasation into blood or lymph vessels [2].

Detection and quantification of circulating tumor cells (CTCs) from blood in the early stages of the disease are important for early diagnosis and treatment. Blood samples potentially containing tumor cells entering the circulation can be evaluated as “liquid biopsy” samples that can generate molecular data about cancer at the single‐cell level [3]. The most common approach for CTC or other peripheral cell isolation is based on differences in the physical and morphological properties, such as shape, structure, form, and size of the cells [4]. However, molecular approaches based on specific surface antigens overexpressed by CTCs are suggested [5]. These surface proteins, which show different levels of expression in cancer cells, are often used as targets in CTC detection methodologies [6].

Biosensors are analytical devices that combine a physicochemical detector with biological components to detect an analyte [7]. Biosensor development and performance largely depend on the optimization of surface functionalization. Biomolecules with specific binding properties, such as antibodies or aptamers, when immobilized on functional surfaces, can improve biosensor performance [8].

Antibodies have been used as ideal capture agents in immunoassay methods due to their high binding affinity and sensitivity for their corresponding antigens [9]. They are immobilized on the solid support surface via carboxyl, amine, hydroxyl, sulfhydryl, alkyl, and aryl functional groups for use in immunodiagnostic approaches [10, 11]. There are various microarray studies using antibodies for signaling pathways, drug mechanisms, clinical research, autoimmune diseases, infectious diseases, neurodegenerative diseases, cancer [12], and CTC capture [13].

On the other hand, the macromolecular structure of antibodies presents some obstacles to decorating better‐defined and more sensitive microarrays. Fab’ (fragment‐antigen‐binding) units, which are antibody fragments produced by enzymatic digestion of intact or recombinant antibodies, are ideal candidates for the development of surfaces with high specificity and sensitivity. Fab’ fragments are smaller molecules than the antibodies from which they derive. The surface‐bound density is higher, and therefore, more antigen‐binding sites are available per unit area on the surface [14, 15]. Fab fragments are most commonly generated by the digestion of intact antibodies using pepsin or papain enzymes. After digestion, F(ab')_2_ dimers are incubated with chemical reductants such as dithiothreitol (DTT), 2‐mercaptoethanolamine (2‐MEA), and TCEP to form Fab’ fragments [16]. Fab’ fragments contain cysteine amino acids with the potential for use as nucleophilic anchors for surface immobilization [17]. Reactive thiol groups on Fab’ fragments enable oriented surface decoration during immobilization, allowing the design of well‐defined microarrays [18, 19].

Detection of CTCs by immunoaffinity in liquid biopsy is an important approach. Epithelial cell adhesion molecule (EpCAM), a 30–40 kDa type I glycosylated membrane protein, is one of the target surface proteins commonly used in immunodiagnostic methods in cancer [20]. Currently, the only FDA‐approved system for CTC detection in liquid biopsy is the antibody‐based immunomagnetic technique, the CellSearch (Veridex) platform. CellSearch captures and detects tumor‐derived cells by targeting the EpCAM molecule [21].

In this study, an immunosurface immobilized with the EpCAM Fab’ fragment, capable of capturing target cells with high sensitivity, was introduced. Intact antibody was fragmented using pepsin proteolytic enzyme, and EpCAM Fab’ fragments were immobilized on a gold surface to detect MCF‐7 human breast cancer cells. The thiol (–SH) groups of Fab’ fragments reacted with gold atoms on the surface, yielding strong thiol‐gold bonds [22]. The morphology of the surfaces was observed with atomic force microscopy (AFM) and scanning electron microscopy (SEM). The number of MCF‐7 cells attached to the surface was observed by fluorescence microscopy. Accurate and sensitive detection of cancer cells in the circulation can be significantly effective in early diagnosis of the disease, prediction of prognosis, and treatment.

## 2 Materials and Methods

### 2.1 Materials

Acrylamide, bisacrylamide, glycine, sodium dodecyl sulfate (SDS), methanol, glacial acetic acid, Coomassie blue R‐250, and phosphate‐buffered saline (PBS) were purchased from Sigma‐Aldrich. The anti‐EpCAM antibody and human IgG antibody were purchased from Santa Cruz. Pepsin from porcine gastric mucosa was purchased from Sigma. Slide‐A‐Lyzer dialysis cassettes (2000 Da MWCO) were obtained from Sigma. The protein ladder was purchased from Invitrogen.

### 2.2 Antibody Fragmentation

Recombinant anti‐EpCAM antibody (0.1 mg/mL) was incubated with pepsin in acetate buffer (pH 4.0) (molar ratio of 1:20, pepsin:antibody) at 37°C for 16 h. In‐solution digestion was stopped by the addition of 2 M Tris base solution to yield a solution with pH = 7.0. F(ab’)_2_ fragment was reduced with 5 mM TCEP at 40°C for 30 min to obtain the Fab’ fragments. Fab’ fragments were characterized by SDS‐PAGE [23]. The analyses were performed using 4%–12% SDS‐PAGE gels at constant voltage (220 V). The molecular weights of proteins were determined using the protein ladder (10–190 kDa). Coomassie blue (0.05% (w/v)) was used to stain the gels, and destaining was performed using a solution containing 40% (v/v) methanol and 10% (v/v) glacial acetic acid in distilled water.

### 2.3 APTES Modification, Gold Coating, and Fab’ Immobilization of Glass Surfaces

The glass surfaces (1 cm × 1 cm) were rinsed with deionized water, followed by ethanol. Then the surfaces were washed with distilled water, ethanol, and acetone for 10 min each. Reactive hydroxyl groups were generated by shaking the surfaces in piranha solution for 10 min and then rinsing with distilled water for 5 min. Surfaces were immersed in 3% APTES in ethanol at room temperature for 2 h [24].

After APTES modification, the glass surfaces were coated with gold using the Bal‐Tec SCD 005 Sputter Coater at 100 mA for 20 s, resulting in an approximate gold thickness of 10–15 nm per deposition cycle. Following gold coating, the surfaces were washed with distilled water and incubated with Fab’ fragments (0.1 mg/mL) overnight at +4°C. After incubation, the surfaces were rinsed with distilled water.

### 2.4 Fourier‐Transform Infrared Spectroscopy (FTIR) Analysis

The APTES modification, gold coating, and Fab’ immobilization on the glass surfaces were characterized using FTIR spectroscopy (Perkin Elmer, USA). Analyses were performed on five different surfaces: bare glass (SiO_2_), hydroxylated glass (SiO_2_‐OH), APTES‐functionalized glass (SiO_2_‐APTES), gold‐coated glass (SiO_2_‐APTES‐Au), and Fab’ immobilized surface (SiO_2_‐APTES‐Au‐Fab’). The absorbance spectra were recorded within the 4000–400 cm^−1^ range.

### 2.5 SEM Analysis

Samples were gold‐coated using a Bal‐Tec SCD005 Sputter Coater at 100 mA current for 20 s before SEM analysis. After the coating process, the surface analyses were performed using an LEO‐1430 VP SEM device at 20 kV. The following surface groups were tested: bare glass (SiO_2_), hydroxylated glass (SiO_2_‐OH), APTES‐functionalized glass (SiO_2_‐APTES), and Fab’ immobilized surface (SiO_2_‐APTES‐Au‐Fab’).

Elemental analysis was performed using SEM EDX to detect Si, N, and C signals. Data were obtained from three randomly selected regions per sample for the representative analysis.

### 2.6 AFM

The topography of the functionalized surfaces was observed by AFM (Park System/XE‐100). AFM measurements were operated in non‐contact mode at room temperature. Surface images were obtained at a frequency of 0.22 Hz using silicon/aluminum‐coated cantilevers (PPP‐NCHR 10M, Park System Ltd., Korea) with a typical force constant of 42 N/m. XEP software (version 1.8.0) was used for data analysis of AFM topographic mappings.

### 2.7 Cell Culture

To test the cell capture efficiency and selectivity of Fab’‐immobilized surfaces, the EpCAM high‐expressing (EpCAM (+)) human breast cancer cell line MCF‐7 (ATCC HTB‐22) and the EpCAM‐negative (EpCAM (‐)) human lymphoblast cell line K562 were used. Cells were cultured in media containing 10% FBS and 1% penicillin/streptomycin at 37°C in 5% CO_2_.

Fab’‐conjugated surfaces prepared via thiol‐gold bonding were tested for cell capture efficiency. Adherent MCF‐7 cells were trypsinized, removed from the flask, precipitated by centrifugation, and suspended in PBS. As K562 cells are non‐adherent, they were directly precipitated by centrifugation and suspended in PBS. The total cell number for both cell lines was adjusted (10,000 cells/mL) using the automated Cell Counter (TC20 Bio‐Rad). Fab’‐immobilized surfaces were washed three times with PBS. Cells (5000 cells in 500 µL PBS) were added onto the surfaces and incubated for 10 min. After incubation, the surfaces were gently washed three times with PBS to remove non‐adherent cells. The captured cells were stained with DNA‐specific dye (DAPI (4’,6‐diamidino‐2‐phenylindole) and visualized under a fluorescent microscope (Zeiss Axio Observer Z1) using Zen Blue Edition software. DAPI was excited using a 365 nm laser.

## 3 Results and Discussion

### 3.1 Production and Characterization of Fab’ Fragments

**FIGURE 1:**
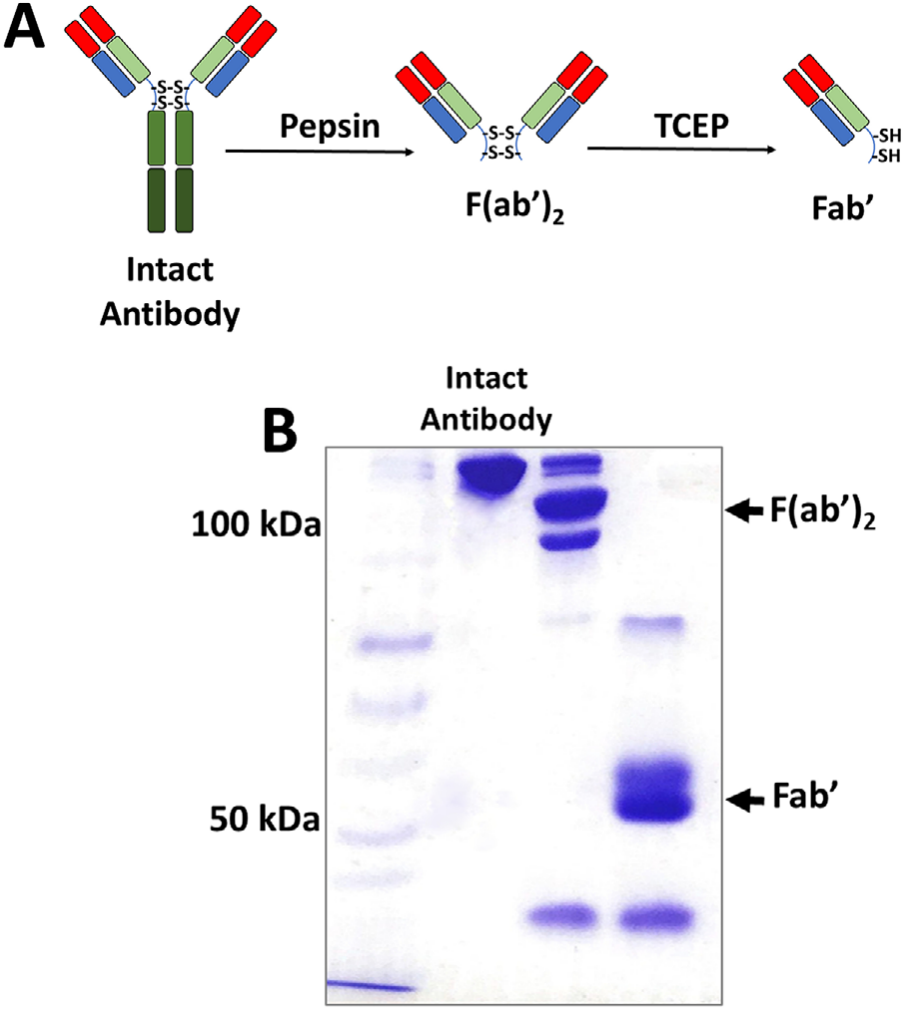
(A) Antibody fragments were obtained by pepsin digestion followed by TCEP reduction. The reduction step generates free thiol (‐SH) groups on Fab’ fragments, which enable site‐specific conjugation to a functional surface. (B) SDS‐PAGE analysis of fragmentation reaction steps. Line 1: protein marker, Line 2: Anti‐EpCAM antibody, Line 3: F(ab’)_2_, Line 4: Fab’.

The general molecular structure of immunoglobulin consists of two polypeptide chains, F(ab’)_2_ fragments, responsible for antigen binding, and an Fc hinge region [25]. Pepsin digests lower sections of the intact antibody below the disulfide bonds located on the heavy chains. The resulting fragment consists of two Fab’ fragments linked by disulfide bonds, which is called F(ab')_2_. F(ab')_2_ can be cleaved into ‐SH‐bearing Fab’ fragments by the reduction of disulfide bonds using TCEP [26]. Fab fragments used as surface elements in this study were obtained from a commercially available anti‐EpCAM antibody. The anti‐EpCAM antibody was digested with pepsin [27] and reduced with TCEP, as described in the method section, following the literature procedure [28, 29]. The formation of Fab’ fragments was illustrated in Figure 1A.

Fragmentation products (F(ab’)_2_ and Fab’ fragments) were analyzed by SDS PAGE. In Figure 1B, the first line shows protein markers, and the second line shows intact anti‐EpCAM antibody with a molecular weight of approximately 150 kDa. In the third line, F(ab’)_2_ fragment was indicated after pepsin digestion, and the molecular weight of the F(ab’)_2_ fragment antibody was around 100 kDa, which is equal to half the molecular weight of the native antibody [15]. In the next step, the F(ab')_2_ portion was reduced by TCEP for the production of the Fab’ fragment, which contains thiol (‐SH) groups and a molecular weight of approximately 50 kDa [30].

Since Fab’ fragments show better surface binding stability and a high density of antigen binding sites on the surface compared to the intact antibody [31, 32, 33, 34], EpCAM antibody fragments were preferred in this study. In addition, gold thiol covalent conjugation occurs without the need for an activating step or catalyst, which makes this reaction feasible for such biosensor production [35]. Fab fragment‐based biosensors are frequently used because Fab’ fragments are rapidly and cheaply produced from intact antibodies via simple reaction steps. Also, Fab’ fragments can be immobilized by covalent conjugation to functional surfaces [36]. This allows for the development of a variety of biosensor strategies by designing different functional surfaces [27].

### 3.2 APTES Modification, Gold Coating, and Fab’ Fragment Immobilization

**FIGURE 2:**
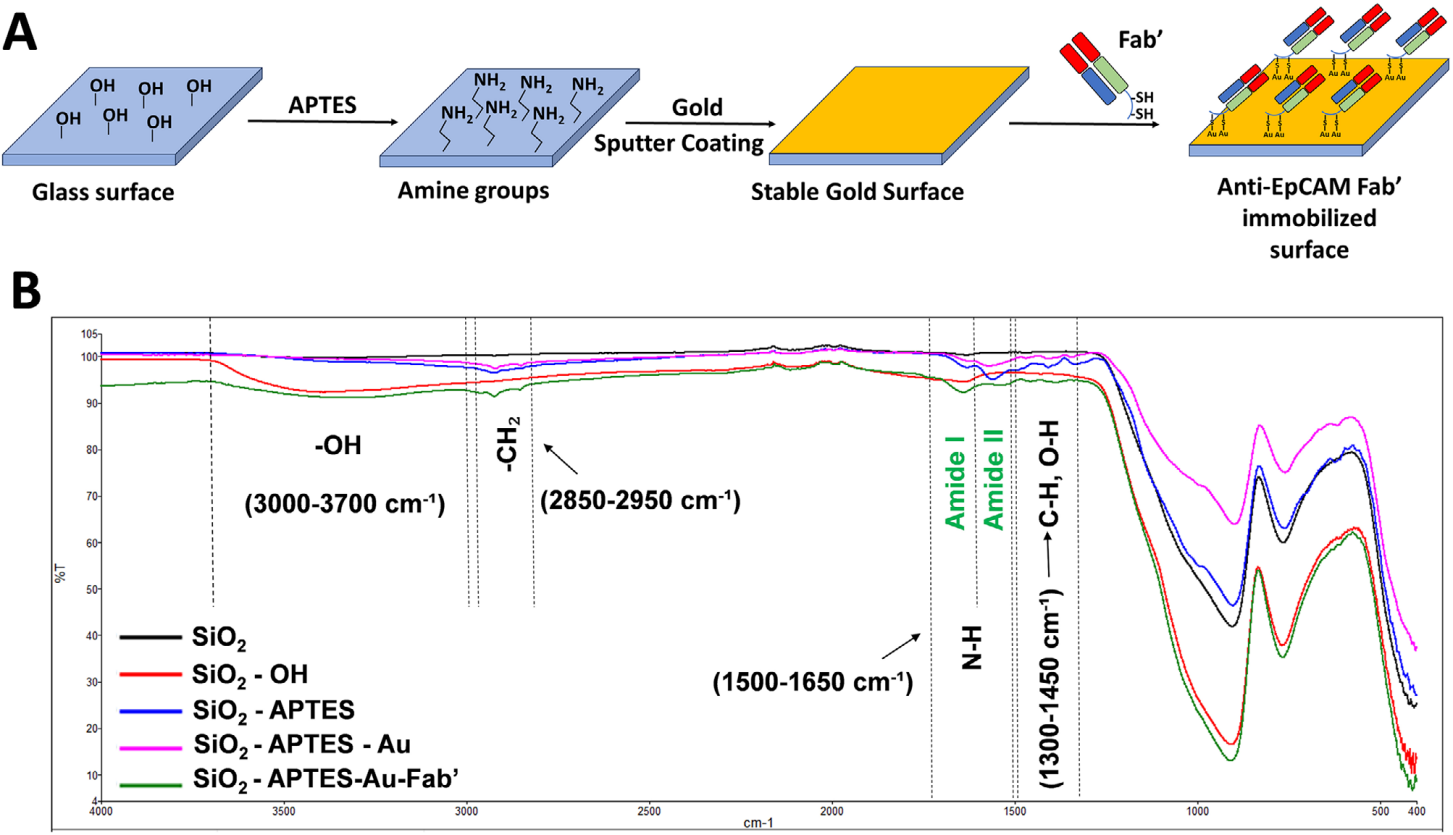
(A) Schematic illustration of the surface modification procedure, including APTES silanization, gold sputter coating, and Fab’ fragment immobilization. (B) FTIR spectra of the surfaces at different modification stages: SiO_2_, SiO_2_–OH, SiO_2_–APTES, SiO_2_–APTES–Au, and SiO_2_–APTES–Au–Fab’.

In this study, a stepwise surface functionalization approach was employed to prepare gold‐coated glass substrates capable of selectively capturing EpCAM‐overexpressing cancer cells. For stable gold coating on glass surfaces, the glass slides (1 cm × 1 cm) were functionalized with APTES and then coated with gold using the Bal‐Tec SCD 005 Sputter Coater, followed by immobilization of Fab’ fragments via a thiol‐selective conjugation reaction. In the reaction, the gold surface breaks the S–H bond, and the deprotonation of thiols produces thiyl radicals that form the Au–S covalent bond [37]. The presence of thiol (–SH) groups on Fab’ fragments enables the formation of strong Au‐S bonds on the surface [38]. A schematic illustration of the surface modification steps is shown in Figure 2A.

To confirm the success of each modification step, Figure 2B displays the FTIR spectra of SiO_2_, SiO_2_–OH, SiO_2_–APTES, SiO_2_–APTES–Au, and SiO_2_–APTES–Au–Fab’ surfaces. An unmodified SiO_2_ surface was used as a control group. A broad absorption band in the range of 3000–3700 cm^−1^ was observed on the SiO_2_–OH surface. This band corresponds to the stretching vibrations of hydroxyl groups (‐OH) on the surface, indicating that the hydroxylation of the SiO_2_ surface was successfully achieved.

The absorption bands in the range of 2850–2950 cm^−1^ observed on the SiO_2_–APTES, SiO_2_–APTES–Au, and SiO_2_–APTES–Au–Fab’ surfaces correspond to the symmetric and asymmetric stretching vibrations of the –CH_2_ groups in the APTES molecule [39, 40]. In addition, the absorbance signals in the range of 1500–1650 cm^−1^ (N‐H symmetric bending) on ​​the SiO_2_‐APTES, SiO_2_‐APTES‐Au, and SiO_2_–APTES–Au–Fab’ surfaces can also be assigned to the NH_2_ group of the APTES molecule [41, 42, 43]. Weak absorption bands observed in the 1300–1450 cm^−1^ range were attributed to vibrations of the C–H and O–H groups in the structure of APTES [44, 45]. The absence of these bands on the unmodified glass surface (SiO_2_) further confirms successful APTES modification.

In their study, Otoufi et al. coated SiO_2_ structures with gold nanoparticles after APTES modification. Through FTIR analysis, they showed a significant decrease in SiO_2_‐related signals after gold nanoparticle coating due to gold coating [46]. In our study, the significant decrease in the vibration signals in the 650–850 cm^−1^ and 850–1250 cm^−1^ ranges, which are attributed to SiO_2_, as well as the characteristic ‐CH_2_ (2850–2950 cm^−1^) and NH_2_ (1500–1650 cm^−1^) signals originating from APTES modification on SiO_2_‐APTES‐Au surfaces, is due to the gold coating stage.

The marked increase in the –NH_2_ signal, which had been attenuated by the gold coating, after Fab’ modification (SiO_2_–APTES–Au–Fab’) provides strong spectral evidence that Fab’ fragments have been successfully immobilized on the surface. This enhancement is particularly associated with the signals observed in the 1500–1650 cm^−1^ range. The signals observed in this region are identified as the Amide I band, arising from the stretching vibrations of carbonyl groups (C = O), and the Amide II band, which results from N–H bending and C–N stretching vibrations [47]. These bands are associated with the contributions of the amine (–NH_2_) and amide (–CONH–) groups present in the structure of Fab to the FTIR spectrum [48, 49, 50].

**FIGURE 3:**
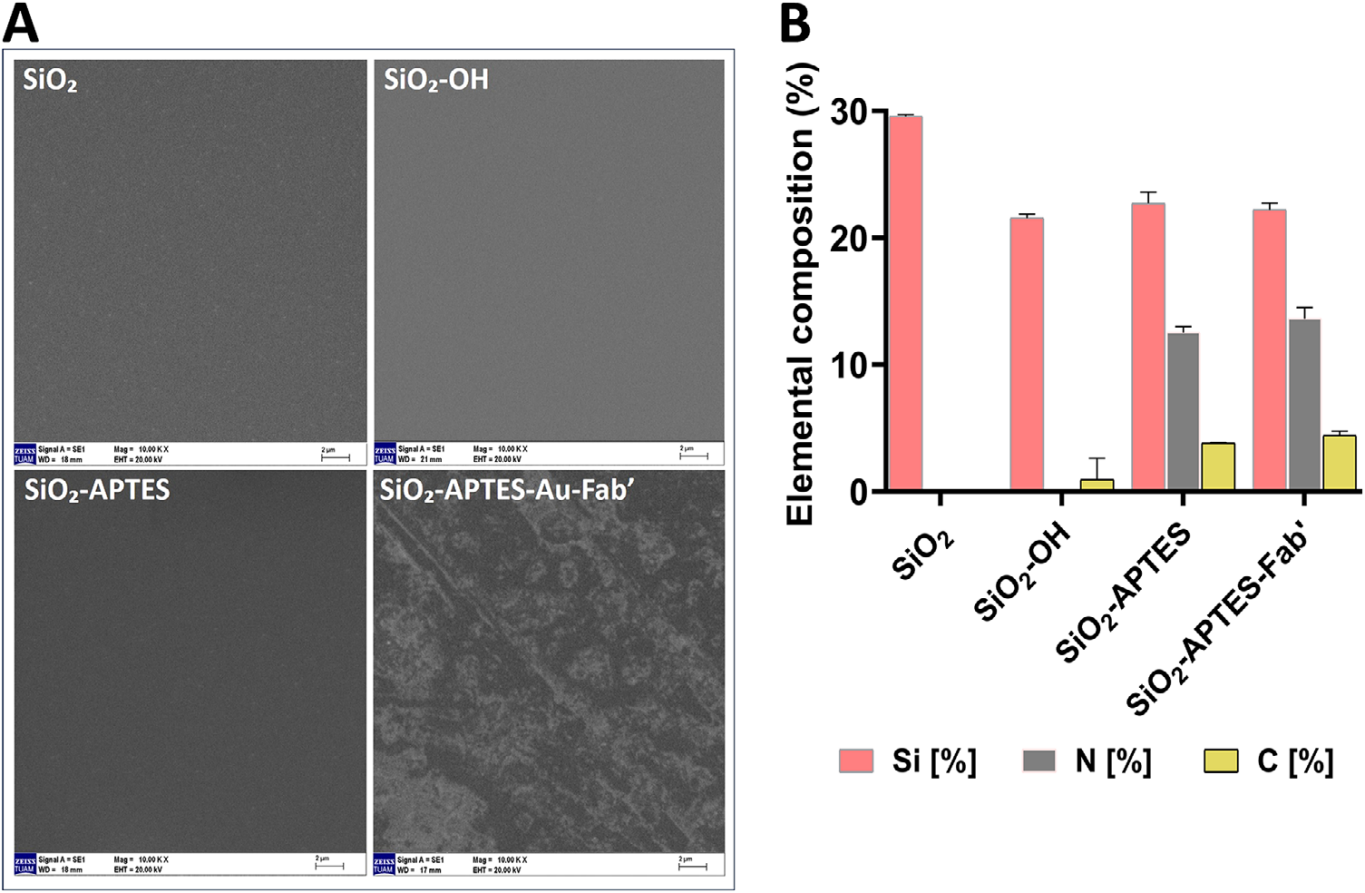
A. SEM images of unmodified glass (SiO_2_), APTES‐functionalized glass (SiO_2_–APTES), and Fab’ immobilized surfaces (SiO_2_–APTES–Au–Fab’) (magnification 10,000×; scale bars: 2 µm). (B) Elemental composition of Si, C, and N detected by EDX analysis at each surface modification step, presented as mean ± SD.

According to SEM images of the SiO_2_, SiO_2_–OH, SiO_2_–APTES, SiO_2_–APTES–Au, and SiO_2_–APTES–Au–Fab’ surfaces, a thin layer of morphological change was observed only after the immobilization of Fab’ fragments onto the SiO_2_–APTES–Au surface (Figure 3A). This observation is consistent with previous studies reporting that antibody or protein conjugation leads to detectable topographic changes on modified surfaces [51]. Such morphological changes are a direct indicator of successful biomolecule immobilization and are critical for sensor performance.

EDX elemental analyses showed an approximately 8% reduction in silicon content after hydroxylation, indicating successful silanization. Higher nitrogen (12.5%) and carbon (3.8%) levels were found on the APTES‐functionalized surface, which is related to the amino groups in the APTES molecules. Further increases in nitrogen (13.6%) and carbon (4.4%) after Fab’ conjugation confirmed the successful attachment of antibody fragments to the gold‐coated surface (Figure 3B). Similar elemental changes have been reported, confirming covalent biomolecule binding on the surface [52].

The stable thiol‐gold interactions on the surface provide the basis for fabricating robust immuno surfaces in both experimental and theoretical studies of biosensor applications [53, 54]. Therefore, the high antigen‐binding capacity and stable adhesion of Fab’ fragments are key advantages expected to enhance the selectivity and sensitivity of the biosensor platform.

**FIGURE 4:**
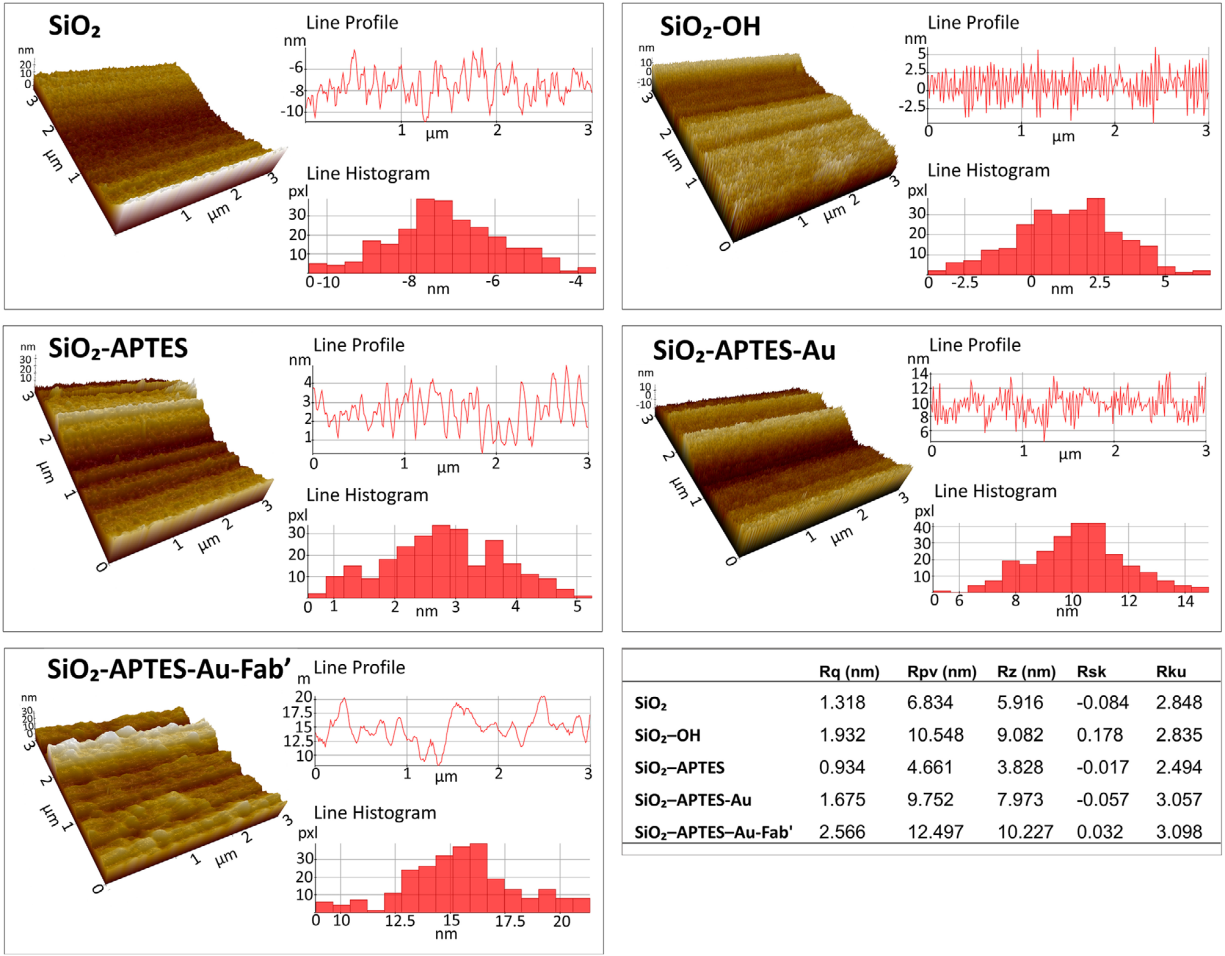
3D AFM topographic images, line profiles, and height distribution histograms of surfaces at each modification step: bare SiO_2_, hydroxylated SiO_2_–OH, silanized SiO_2_–APTES, gold‐coated SiO_2_–APTES–Au, and Fab’‐immobilized SiO_2_–APTES–Au–Fab’. Surface roughness parameters were summarized in the table. Parameters include root mean square roughness (Rq, nm), peak‐to‐valley height (Rpv, nm), average maximum height (Rz, nm), surface skewness (Rsk), and kurtosis (Rku). Scan range was 0–1000 nm.

AFM analysis, including both topographical images and quantitative roughness parameters, revealed distinct surface changes at each modification step (Figure 4). The bare SiO_2_ surface showed a smooth and uniform topography (Rq = 1.318), with a relatively low peak‐to‐valley height (Rpv = 6.834) and minimal asymmetry in height distribution (Rsk = –0.084). The line profile and histogram of the SiO_2_ image support this, indicating a narrow distribution centered around –7 to –8 nm. Surface roughness increased following hydroxylation (SiO_2_–OH) (Rq = 1.932 nm) with a peak‐to‐valley difference of 10.548 nm, indicating successful surface activation. The Rsk value became slightly positive (0.178), and the AFM image shows a rougher surface with a histogram centered around ±5 nm. The silanized glass surface exhibited the lowest Rq (0.934 nm), consistent with a dense and homogeneous silane monolayer, supported by a symmetric line profile (Rsk = −0.017) and low kurtosis (Rku = 2.494). Joshi et al. [55] reported that APTES modification under anhydrous conditions resulted in a more uniform, cluster‐free, and mechanically stable silane layer compared to that formed under aqueous conditions (Rq ∼0.5 nm vs. 1.4 nm). Gold coating (SiO_2_–APTES–Au) significantly increased surface heterogeneity, with Rq rising to 1.675 nm and Rpv to 9.752 nm. Finally, the Fab’‐immobilized surface exhibited the highest roughness and heterogeneity (Rq = 2.566 nm, Rpv = 12.497 nm, Rz = 10.227 nm). The AFM image shows the coarsest surface, and the histogram is broad, ranging from 12 to 17 nm. The positive Rsk (0.032) and high Rku (3.098) further confirm the significant surface modification resulting from Fab’ attachment. AFM data indicate that surface roughness increases after the immobilization of large biomolecules such as Fab’. In addition, steric factors, such as the size and hydration state of the adsorbed molecules, influence how these molecules arrange themselves on the surface and affect the morphology observed by AFM [56]. The evenness of protein immobilization and the uniform structure of the silane layer can also impact the overall surface topography [55].

While intact antibodies (150 kDa) typically appear with sizes of 8–10 nm in AFM topographic images [57], the Fab’ fragment (∼50 kDa) measures approximately 2–3 nm. In our study, the Fab’‐immobilized surface exhibited significantly higher roughness compared to other groups (Rq = 2.566 nm), with a broad distribution ranging from 12 to 17 nm observed in the histogram. In immunocapture applications, the use of smaller agents, such as Fab’, increases the binding surface area and reduces steric hindrance, thereby facilitating access to target molecules [53, 58].

Lee et al. developed a biosurface by immobilizing anti‐insulin Fab’ fragments on gold surfaces via Au‐S covalent bonds. Specifically bound bovine insulin was successfully detected at concentrations ranging from 100 ng/mL to 10 µg/mL on the designed biosurface [30]. In another study using an SPR‐based biosensor, anti‐morphine Fab’ fragments were covalently bound to gold surfaces through free thiol groups. These fragments, containing antigen‐binding sites, showed a high affinity to morphine [59].

Fab’ fragments can be attached to Au regions via thiol groups by covalent conjugation. Based on this principle, Salmonella detection was performed using SPR sensors by immobilizing TCEP‐reduced Fab’ fragments via free thiol groups. The biosensor detected bacterial concentrations from 10^3^ to 10⁸ CFU/mL with high sensitivity [38]. Similarly, the Fab’‐based immunosensor demonstrated higher sensitivity and faster analysis using SPR [60]. Fab’ fragments, which have a higher capture ability than intact antibodies, generally bind directly to gold surfaces via thiol groups and form stable surfaces as mentioned in the literature examples above. Therefore, the same strategy was used in our study.

To further confirm the successful immobilization of Fab’ fragments on the gold‐coated surfaces, the concentration and binding efficiency were quantified using a bicinchoninic acid (BCA) assay. A significant increase in Fab’ binding was observed on the SiO_2_–APTES–Au–Fab’ surfaces compared to control surfaces without Fab’ (*p* < 0.001), with an estimated Fab’ concentration of approximately 29 µM. These results complement the FTIR, SEM, and AFM findings and confirm effective Fab’ immobilization (Figure S2).

### 3.3 The Attachment Study of EpCAM (+) and EpCAM (−) Cells

**FIGURE 5:**
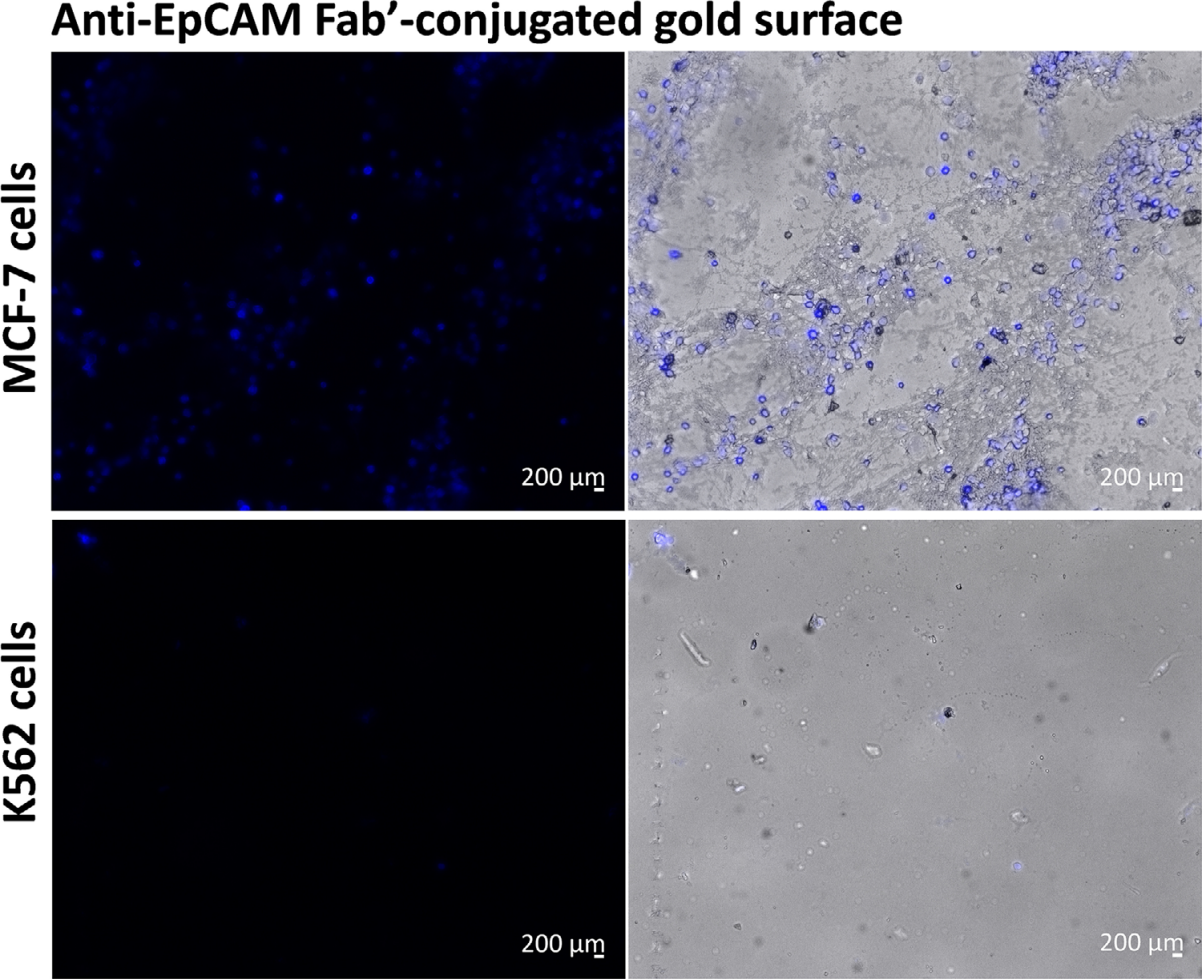
Fluorescence and merged brightfield images of MCF‐7 and K562 cells on gold surfaces conjugated with anti‐EpCAM Fab’ fragments. Each cell type is shown with DAPI fluorescence images on the left and the merged brightfield and fluorescence images on the right. Scale bar: 200 µm.

In the study, the EpCAM antibody Fab’ fragment was immobilized on a gold surface and evaluated for its selective and efficient capture of CTCs. For functionalized surface capturing experiments, live cells were used. Fluorescent microscopy analysis showed that MCF‐7 cells, which highly express EpCAM, bound effectively to the Fab’‐coated gold surface, whereas K562 cells, which do not express EpCAM, showed no significant binding (Figure 5). To assess non‐specific adhesion, IgG Fab’ fragments were immobilized on gold‐coated surfaces via thiol‐gold interactions and tested with both MCF‐7 and K562 cell lines. Fluorescence microscopy images (Figure S1B) revealed that neither cell type exhibited any significant binding to the IgG Fab’‐coated surfaces. These findings confirm the specificity of the EpCAM Fab’‐based capture system by demonstrating the absence of non‐specific binding when IgG Fab’ fragments are used as a control.

In many studies, sensitive biosensor technologies are combined with cell‐selective and specific capture techniques [61, 62, 63]. Antibody‐antigen interactions are widely used to reduce heterogeneity of biological samples and enhance biosensor specificity [64, 65]. A microfluidic platform integrated with EpCAM antibody‐coated microposts (“CTC‐chip”) was developed, enabling efficient and selective separation of CTCs. CTC analysis was successfully performed (99% efficiency) in peripheral whole blood samples from patients with metastatic lung, prostate, pancreatic, breast, and colon cancers [66, 67].

EpCAM overexpression has been demonstrated in many human breast cancer cell types [68]. These changes in expression levels, when compared to healthy cells, make the EpCAM receptor an important prognostic marker for cancer therapy [69, 70]. In breast cancer diagnostic studies, CTC detection methods using EpCAM antibodies coupled to immunomagnetic beads have been proposed for the phenotyping or isolation of cancer cells [71]. Immunocapture of the EpCAM‐overexpressing cells is frequently employed to detect and characterize CTC from whole blood [72]. In a study, EpCAM antibody immobilization strategies on functional surfaces were used to evaluate CTC capture effects on MCF‐7 (EpCAM (+)) breast cancer and CCRF‐CEM (EpCAM (‐)) acute lymphoblastic leukemia cells. For this purpose, researchers demonstrated antibody immobilization via EDC‐NHS reaction in a microfluidic channel or via streptavidin attachment using a glutaraldehyde linker, achieving high capture efficiency and selectivity [73].

Recently, Fab’ units have been shown as ideal candidates that can be applied with current immobilization techniques in the development of biosensors with high specificity and sensitivity [74]. In the study, the cell attachment properties of the Fab’ fragment‐immobilized and unconjugated gold surfaces were tested by fluorescent microscope using two different cancer cell lines: a well‐characterized MCF‐7 breast carcinoma cell line, known to express high levels of EpCAM [75], and the K562 cell line, which is EpCAM‐negative [76]. The active sites where the intact EpCAM antibody captures cancer cells are located on the Fab’ fragments. Since the Fab’ fragment is a relatively smaller molecule, it provides a higher surface density, allowing more antigen binding sites per unit area on the surface. In studies comparing the affinities of antibodies and Fab’ fragments against various biological targets in bioactive surface applications, it was observed that Fab’ fragments exhibit significantly higher affinity [56].

## 4 Concluding Remarks

We demonstrated functionalization with APTES, followed by gold coating on glass slides. EpCAM Fab’ fragment was immobilized via thiol groups on the gold‐coated surface, and its functionality was confirmed through cell capture experiments. Fluorescent microscope analysis proved that the EpCAM Fab’ fragment exhibits a highly selective binding affinity for EpCAM‐overexpressing breast cancer cells (MCF‐7) compared to K562 (EpCAM (‐)) cells. This design enables high‐quality CTC detection of rare cell populations in the circulation. The detection of circulating tumor cells plays a critical role in cancer prognosis, early diagnosis, and the monitoring of disease progression, which may vary among individuals.

## Conflicts of Interest

The authors declare no conflicts of interest.

## Supporting information




**Supporting file 1:** elsc70043‐sup‐0001‐SuppMat.pdf

## Data Availability

The data that support the findings of this study are available from the corresponding author upon reasonable request.
