# Supplementary material for: Fab’ Fragment‐Immobilized Gold Surface for Capturing EpCAM‐Positive Breast Cancer Cells
Source: Eng Life Sci. 2025 Sep 12;25(9):e70043. doi: 10.1002/elsc.70043 (PMC12426906; doi:10.1002/elsc.70043)
Supplement: Supplementary file 1 — Supporting file 1: elsc70043‐sup‐0001‐SuppMat.pdf [file ELSC-25-e70043-s001.pdf]

Supplementary

Methods

1.Non-specific IgG Antibody Fragmentation

The same fragmentation protocol used for anti-EpCAM antibody was applied to IgG for preparing IgG Fab fragments. Briefly, IgG antibody (0.1 mg/mL) was incubated with pepsin in acetate buffer (pH 4.0) at a molar ratio of 1:20 (pepsin: antibody) at 37°C for 16 hours. The digestion was stopped by adding a 2 M Tris base solution to adjust the pH to 7.0. The resulting $F(ab')_2$ fragments were reduced with 5 mM TCEP at 40°C for 30 minutes to obtain Fab' fragments. Fab' fragment formation was confirmed by SDS-PAGE analysis. The electrophoresis was performed using 4–12% gradient gels at a constant voltage of 220 V. Protein molecular weights were estimated using a 10–190 kDa protein ladder. Gels were stained with 0.05% Coomassie blue and destained with a solution containing 40% methanol and 10% glacial acetic acid in distilled water.

2. Fluorescence Microscope Imaging

IgG Fab' fragments immobilized on gold-coated surfaces via thiol-gold bonding were used as a non-specific binding control in cell adhesion experiments. Adherent MCF-7 cells were detached using trypsin, collected by centrifugation, and resuspended in PBS. Non-adherent K562 cells were directly collected by centrifugation and resuspended in PBS. Cell concentrations for both lines were adjusted to 10,000 cells/mL using an automated cell counter (TC20 Bio-Rad). IgG Fab'-coated surfaces were washed three times with PBS. Then, 5,000 cells in 500 µL PBS were added onto each surface and incubated for 10 minutes. After incubation, surfaces were gently washed three times with PBS to remove non-adherent cells. Captured cells were stained with DAPI (4',6-diamidino-2-phenylindole) and visualized under a Zeiss Axio Observer Z1 fluorescence microscope using Zen Blue Edition software. DAPI excitation was performed with a 365 nm laser.

3. BCA Assay for EpCAM Fab' Immobilization and Quantification

To assess the concentration and binding efficiency of EpCAM Fab' fragments on the functionalized surfaces, a bicinchoninic acid (BCA) protein assay was performed using the Pierce BCA Protein Assay Kit (Thermo Scientific, USA). Functionalized surfaces (1 cm x 1 cm), including SiO2, SiO2-OH, SiO2-APTES, SiO2-APTES-Au, and SiO2-APTES-Au-Fab', were rinsed with deionized water and dried. Next, 1 mL of BCA reagent was added to each surface and incubated at 37°C for 30 minutes. After incubation, the supernatant was collected, and absorbance was measured at 562 nm using a spectrophotometer (Shimadzu UV-1800). Absorbance values were measured in triplicate (n=3), and results are expressed as mean ± SD.

Statistical analyses were performed using GraphPad Prism 8 software Two-way ANOVA was performed to compare the binding amounts between Fab' fragment-immobilized SiO_2–APTES–Au–Fab' surfaces and SiO_2–APTES–Au control surfaces. Statistical significance was determined at $p < 0.01$.

Results

1. SDS PAGE Analysis of Non-Specific IgG Antibody Fragments

Fragmentation products of IgG ($F(ab')_2$ and Fab' fragments) were analyzed by SDS-PAGE (Supplementary Figure 1A). In lane 1, protein molecular weight markers ranging from 10 to 190 kDa were shown. Lane 2 corresponds to the intact IgG antibody, which migrated at approximately 150 kDa. After pepsin digestion, a distinct band corresponding to the $F(ab')_2$ fragment (~100 kDa) was observed in lane 3. Subsequent reduction with TCEP produced Fab' fragments, which appeared as a lower molecular weight band (~50 kDa) in lane 4, confirming successful fragmentation and reduction.

2. IgG Fab' Fragments Immobilized on Surface Cells Capture Test

To assess non-specific cell adhesion, IgG Fab' fragments were immobilized on gold-coated surfaces via thiol-gold interactions and tested using MCF-7 (EpCAM-positive) and K562 (EpCAM-negative) cell lines. Fluorescence microscopy images (Supplementary Figure 1B) revealed that neither MCF-7 nor K562 cells exhibited any significant binding to the IgG Fab'-coated surfaces. These findings confirm the specificity of the EpCAM Fab'-based capture system by demonstrating that non-specific binding is absent when IgG Fab' fragments are used as a control.

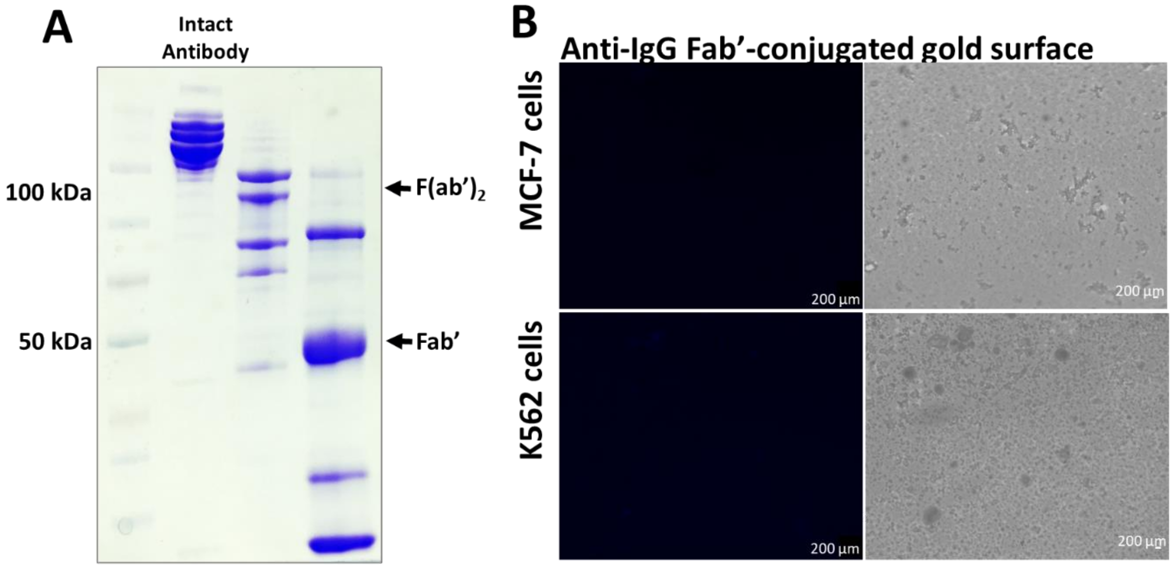

Supplementary Figure 1. (A). SDS-PAGE analysis of IgG Fab' fragmentation reaction steps. Line 1: protein marker, line 2: intact IgG antibody, line 3: $F(ab')_2$, line 4: Fab'. (B) Fluorescence and merged brightfield images of K562 and MCF-7 cells on gold surfaces conjugated with IgG Fab' fragments. Each

cell type is shown with DAPI fluorescence images on the left, and the merged brightfield and fluorescence images on the right. Scale bar: 200 μm.

3.The Concentration and Binding Efficiency of EpCAM Fab' Fragments

The concentration and binding efficiency of Fab' fragments immobilized on the functionalized surfaces were evaluated using the BCA protein assay (**Supplementary Figure 2**). A statistically significant difference in binding amount was observed between the Fab' fragment-immobilized SiO_2–APTES–Au–Fab' surfaces and the SiO_2–APTES–Au surfaces ($p < 0.001$). Based on the standard curve, the amount of Fab' fragments bound to the SiO_2–APTES–Au–Fab' surface was estimated to be approximately 29 μM. These results confirm the effective immobilization of Fab' fragments on the gold-coated functionalized surface.

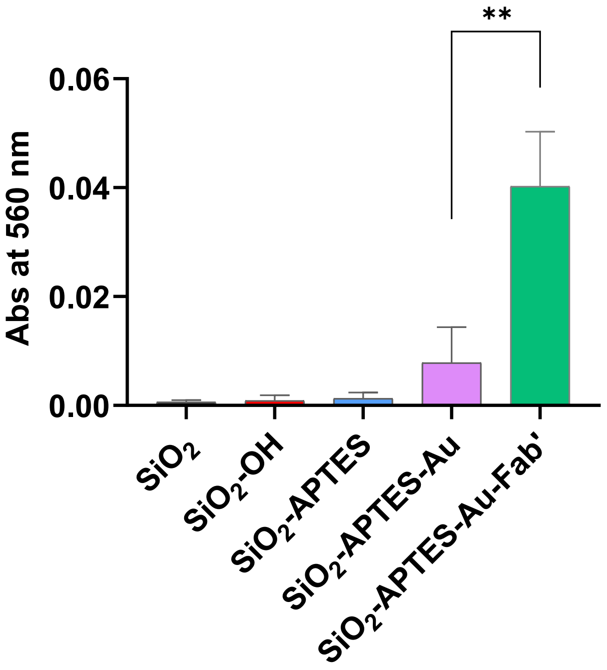

Supplementary Figure 2. Spectrophotometric analysis of immobilized EpCAM-Fab' on functionalized surfaces.
